# mTORC1 Deficiency Prevents the Development of MC903-Induced Atopic Dermatitis through the Downregulation of Type 2 Inflammation

**DOI:** 10.3390/ijms24065968

**Published:** 2023-03-22

**Authors:** Anupriya Gupta, Keunwook Lee, Kwonik Oh

**Affiliations:** 1 Department of Pathology, College of Medicine, Hallym University, Chuncheon 24252, Republic of Korea; 2 Department of Biomedical Science, Hallym University, Chuncheon 24252, Republic of Korea; 3 Institute of Medical Science, College of Medicine, Hallym University, Chuncheon 24252, Republic of Korea

**Keywords:** atopic dermatitis, dimethyloxalylglycine (DMOG), hypoxia inducible factor (HIF), MC903, mTORC, TSLP

## Abstract

Atopic dermatitis (AD) is a chronic inflammatory skin disease characterized by eczema and itching. Recently, mTORC, a central regulator of cellular metabolism, has been reported to play a critical role in immune responses, and manipulation of mTORC pathways has emerged as an effective immunomodulatory drug. In this study, we assessed whether mTORC signaling could contribute to the development of AD in mice. AD-like skin inflammation was induced by a 7-day treatment of MC903 (calcipotriol), and ribosomal protein S6 was highly phosphorylated in inflamed tissues. MC903-induced skin inflammation was ameliorated significantly in Raptor-deficient mice and exacerbated in Pten-deficient mice. Eosinophil recruitment and IL-4 production were also decreased in Raptor deficient mice. In contrast to the pro-inflammatory roles of mTORC1 in immune cells, we observed an anti-inflammatory effect on keratinocytes. TSLP was upregulated in Raptor deficient mice or by rapamycin treatment, which was mediated by hypoxia-inducible factor (HIF) signaling. Taken together, these results from our study indicate the dual roles of mTORC1 in the development of AD, and further studies on the role of HIF in AD are warranted.

## 1. Introduction

Atopic dermatitis (AD) is one of the chronic inflammatory skin diseases characterized by recurrent eczematous lesions and intense itching [1,2,3]. The pathophysiology is complicated and includes exaggerated type 2 inflammation driven by type 2 T helper (TH2) and innate lymphoid cells (ILC2s), and impaired barrier functions include a loss-of-function mutation in the gene encoding filaggrin [4]. These drivers can also promote and interact with each other. For example, skin barrier impairment caused by filaggrin deficiency promotes T-cell infiltration and bacterial infection in the skin. Local type 2 inflammation further diminishes barrier function, causing itching and inducing tissue damage. Some individuals with AD suffer from a series of other allergic diseases, such as allergic rhinitis, food allergies, and asthma, which is the so-called ‘atopic march’ [5]. AD steadily increases in incidence and has been a burden for healthcare resources. However, the development of new therapeutics remains an unmet need.

MC903 (calcipotriol) is an active vitamin D analog without affecting calcium metabolism [6] and has been used for psoriasis patients [7]. In some psoriasis patients, MC903 induces irritant skin inflammation as a side effect. However, in mouse keratinocytes, it upregulates TSLP, which induces AD-like type 2 skin inflammation [8,9]. Since TSLP receptors are expressed in various immune cells, including ILC2s, the TSLP-TSLP receptor interaction activates ILC2s to produce type 2 cytokines, such as IL-4, IL-5, and IL-13 [10] and stimulates eosinophils [11], basophils [12,13], and dendritic cells [14], which eventually causes AD-like skin inflammation. Collectively, the MC903-induced skin inflammation model allows us to analyze the roles of various immune cells during the development of AD.

The mechanistic target of rapamycin (mTOR) is a crucial regulator of cellular metabolism and is implicated in cancers and diabetes. mTOR is a serine/threonine kinase, and its signaling proceeds via two complexes: mTOR Complex 1 (mTORC1) and mTORC2 [15,16]. mTORC1 contains Rheb (a small GTPase), the regulatory-associated protein of mTOR (Raptor), G protein β-subunit-like protein (GβL), and the proline-rich Protein Kinase B (PKB)/Akt substrate 40 kDa (PRAS40), and it is rapamycin-sensitive. mTORC1 activation leads to the phosphorylation and activation of the ribosomal S6 kinase and is thought to be associated with ribosome biogenesis, autophagy, and protein translation. mTORC2 contains, in addition to mTOR and GβL, the rapamycin-insensitive companion of mTOR (Rictor) and mammalian stress-activated protein kinase interacting protein-1 (mSin1). The phosphorylation of Akt is one of the downstream targets of mTORC2 [17,18]. Both mTORC1 and mTORC2 are also closely involved in immune responses as well as metabolism. 

mTORC1 also promotes a shift in glucose metabolism from oxidative phosphorylation to glycolysis by increasing the translation of transcription factor hypoxia-inducible factor (HIF), which drives the expression of several glycolytic enzymes [15]. HIF is independently regulated by both mTORC1 and hypoxia. In the presence of sufficient oxygen, HIF is hydroxylated by iron-dependent prolyl hydroxylases (PHDs) and then degraded. Under hypoxia, the activity of PHDs is inhibited, and then HIF is stabilized, allowing translocation to the nucleus and activation of a group of genes that minimize oxygen consumption and restore oxygen delivery [19].

In this study, we found that ribosomal protein S6 was phosphorylated in keratinocytes and inflammatory cells after MC903 treatment, which led us to investigate the roles of mTORC in the MC903-induced AD model. MC903-induced skin inflammation was significantly reduced in the Raptor deficient or rapamycin-treated mice, accompanied by downregulation of IL-4 and reduced numbers of eosinophils, suggesting that mTORC1 is essential for the type 2 skin inflammation induced by MC903. In contrast, TSLP was upregulated in the Raptor deficient or rapamycin-treated mice and downregulated by DMOG (dimethyloxalylglycine). Considering that DMOG inhibits PHD and stabilizes HIF, these findings demonstrate both pro- and anti-inflammatory roles of mTORC1 in the development of AD. 

## 2. Results

### 2.1. Ribosomal Protein S6 Is Phosphorylated in MC903-Induced Skin Inflammation

**Figure 1:**
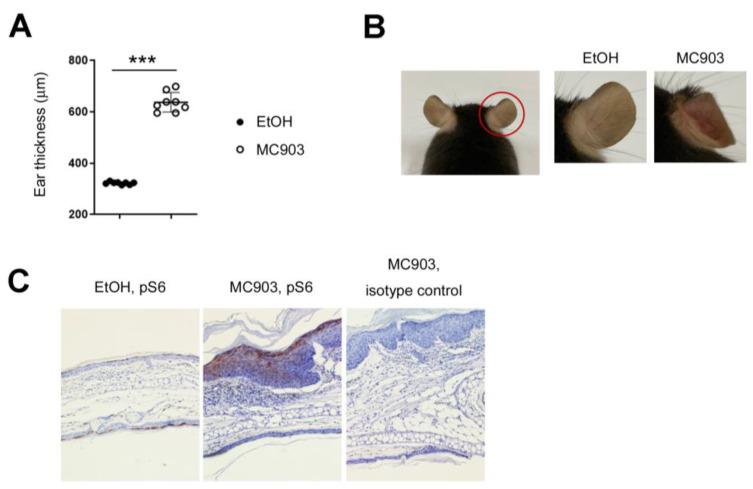
MC903 induced skin inflammation, accompanied by phosphorylation of S6. (**A**) Mice were sensitized on both ears with MC903 (2 nmol in 10 μL of EtOH in each ear) for 7 days. We checked the ear thickness every 3 days and analyzed it on day 7. The ear thickness results on day 7 are shown. Each circle represents a single mouse. (**B**) Gross appearance of the ears after treatment of MC903 for 7 days. (**C**) Immunohistochemical analysis. The skin tissues were stained with anti-pS6 antibodies. A brown color means pS6 positive (×100). Data are presented as the mean ± SD. ***, *p* < 0.001.

**Figure 2:**
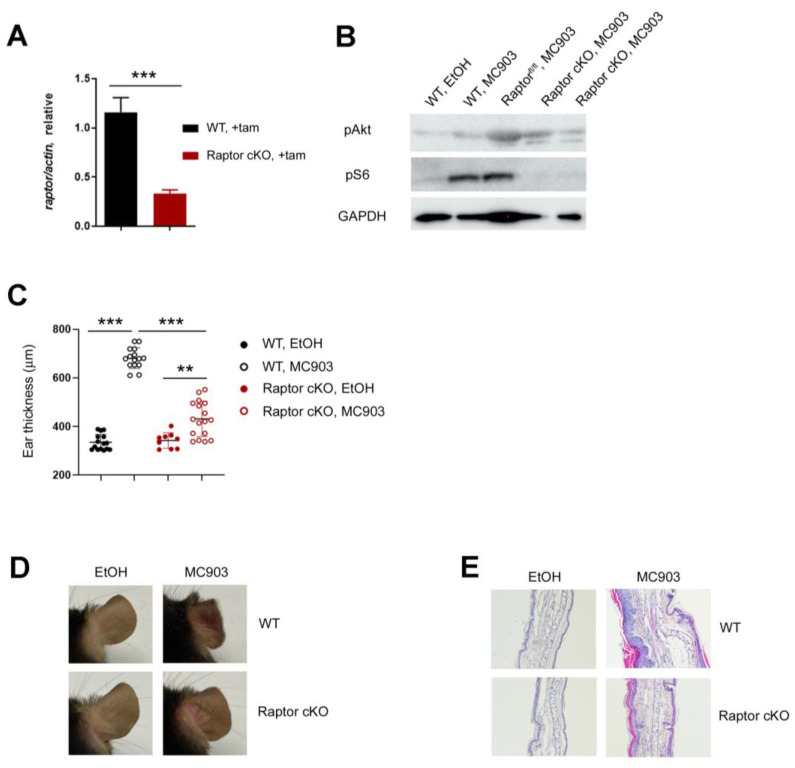
mTORC1 is essential for MC903-induced skin inflammation. (**A**) qPCR shows mRNA expression of Raptor after tamoxifen treatment. (**B**) The expression of pAkt and pS6 in the ear skin of WT and Ert2Cre-*Raptor*^fl/fl^ mice treated with EtOH or MC903. All mice except *Raptor*^fl/fl^ mice were treated with tamoxifen before MC903 treatment. *Raptor*^fl/fl^ mice were treated with the vehicle. (**C**) Ear thickness results are shown. Each circle represents a single mouse. Data are presented as the mean ± SD. **, *p* < 0.01; ***, *p* < 0.001. (**D**) Gross appearance of the ears of WT and Raptor cKO mice after MC903 treatment. (**E**) Histological analysis. Ear tissues were fixed and stained with hematoxylin and eosin ×100).

MC903 has been known to induce type 2 skin inflammation and develop human AD-like symptoms in mice [8,9]. First, we tested the effect of MC903 on the development of AD-like skin inflammation in our mouse facility. 2 nmol of MC903 was applied to the ears every day, and the extent of inflammation was monitored by ear thickness. The ears swelled gradually and became more than twice as thick after 7 days of treatment with MC903 compared with those treated with the vehicle (EtOH) (Figure 1A). The inflamed ears also displayed reddening, swelling, and scaling, and the epidermis began to peel when the treatment period was extended to 14 days (Figure 1B). Histological analysis revealed epidermal hyperplasia and inflammatory cell infiltrates (Figure 1C). We also tested the phosphorylation of ribosomal protein S6 (pS6) using immunohistochemistry to check the activity of mTORC1 and found that both keratinocytes and dermal inflammatory cells were pS6 positive (Figure 1C), suggesting that mTORC1 was activated in both immune cells and keratinocytes. A similar finding was observed in the immunoblotting assay as well (Figure 2B). The serine residue of Akt was also phosphorylated modestly in the skin tissues treated with MC903 (Figure 2B).

### 2.2. mTORC1 Is Essential for MC903-Induced Skin Inflammation

Since the roles of mTORC1 and mTORC2 in type 2 inflammation and AD were controversial [20,21,22,23,24,25], we investigated this issue in the MC903 skin inflammation model using Raptor and Rictor deficient mice. To exclude the potential effects of mTORC1 on the development, we inactivated Raptor in adult mice using the tamoxifen-inducible Ert2Cre transgene. We applied tamoxifen into the ears of Ert2Cre-*Raptor*^fl/fl^ (referred to as Raptor cKO) daily for 5 days and checked the expression of Raptor in the ear tissues using RT-qPCR. After tamoxifen treatment, the floxed Raptor alleles were deleted, and the expressions of the Raptor transcripts (Figure 2A) and the S6 phosphorylation (Figure 2B) were reduced significantly (without tamoxifen, the level of pS6 was comparable between WT and Ert2Cre-*Raptor*^fl/fl^ mice (Figure 2B)). Next, 2 nmol of MC903 was applied to the WT and Raptor cKO mice for 7 days, and the extent of ear swelling was monitored. Although the ears of both WT and Raptor cKO mice swelled after MC903 treatment, the ears of WT mice were much thicker than those of Raptor cKO (Figure 2C). Consistent with the ear thickness results, the signs of inflammation, such as reddening, scaling (Figure 2D), epidermal hyperplasia, and inflammatory cell infiltration were much less severe in the Raptor cKO mice (Figure 2E). 

**Figure 3:**
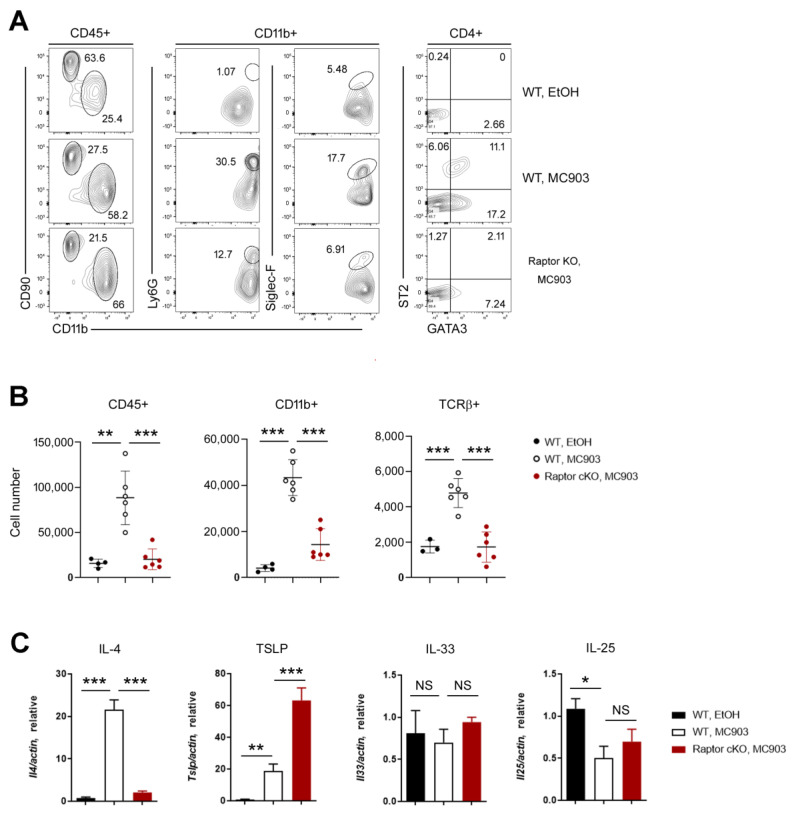
Immunological analysis of the MC903-treated skin tissues. (**A**) FACS analysis of the ears in MC903-induced inflammation. The populations of lymphoid cells (CD90+) and myeloid cells (CD11+) were analyzed in CD45+ populations. The neutrophils (Ly6G+) and eosinophils (SiglecF+) were analyzed in CD11b+ innate cell populations. The expressions of GATA3 and ST2 were analyzed in CD4+ populations. The percentage of each population is shown. (**B**) Cell numbers of hematopoietic cells (CD45+), innate cells (CD11b+), and αβ T cells (TCRβ+) in WT and Raptor cKO ears. (**C**) qPCR shows the mRNA expression of IL-4, TSLP, IL-33, and IL-25 in ears. Data are presented as the mean ± SD. NS, not significant; *, *p* < 0.05; **, *p* < 0.01; ***, *p* < 0.001.

We next investigated the immunologic changes in the ears using flow cytometry. In control WT mice (treated with EtOH), the frequencies of lymphoid (CD90+) and myeloid (CD11b+) cell populations were similar, or there were more lymphoid cells. In contrast, the frequency of myeloid cells, including neutrophils (CD11b + Ly6G+) and eosinophils (CD11b + Siglec-F+), increased dramatically in the MC903-treated WT ears (Figure 3A, left). In lymphoid cell populations, MC903 treatment upregulated TH2 markers, including GATA3 and ST2 in CD4+ T cells (Figure 3A, right) and TH2 cytokines, such as IL-4 (Figure 3C). We also calculated the absolute numbers of each cell population and found that there were more hematopoietic cells (CD45+), myeloid cells (CD11b+), and TCRβ+ T cells in the MC903-treated WT ears (Figure 3B). However, the expression levels of GATA3 and ST2 in CD4+ T cells (Figure 3A) and the numbers of all subsets of inflammatory cells (Figure 3B) were reduced in Raptor cKO mice, supporting that mTORC1 was essential for the activation of immune cells in skin inflammation induced by MC903.

Since the ribosomal protein S6 was phosphorylated in not only immune cells but also keratinocytes (Figure 1C), we checked the expressions of epidermal cell-derived cytokines such as TSLP, IL-33, and IL-25 [21,22]. The expressions of IL-33 and IL-25 were not changed significantly in WT and Raptor cKO. By contrast, TSLP was upregulated by MC903 treatment in WT and even more in Raptor cKO (Figure 3C). To confirm the above results, we repeated the MC903 experiments again using different mouse models.

### 2.3. Rapamycin Prevents Type 2 Inflammation

**Figure 4:**
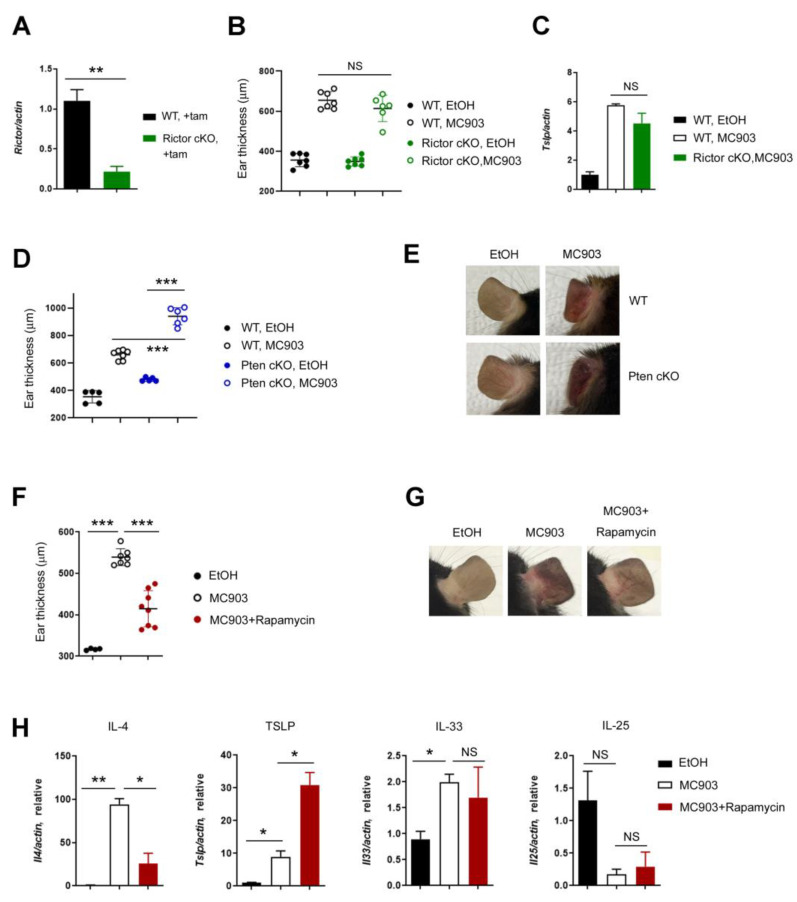
Rapamycin reduced inflammation induced by MC903. (**A**) qPCR shows mRNA expression of Rictor after tamoxifen treatment. (**B**) The difference in ear thickness between WT and Rictor cKO. (**C**) qPCR shows the mRNA expression of TSLP between WT and Rictor cKO after MC903 treatment. (**D**) The difference in ear thickness between WT and Pten cKO. (**E**) Gross appearance of the ears of WT and Pten cKO mice after MC903 treatment. (**F**) The difference in ear thickness between MC903 and MC903 plus rapamycin. (**G**) Gross appearance of the ears of WT mice treated with rapamycin. (**H**) qPCR shows the mRNA expression of IL-4, TSLP, IL-33 and IL-25 in ears. Data are presented as the mean ± SD. NS, not significant; *, *p* < 0.05; **, *p* < 0.01; ***, *p* < 0.001.

It was reported that mTORC2 and its downstream molecules, such as Akt and cathepsin H were essential for the barrier function of the skin, and the disruption of this axis was associated with AD [26], which led us to investigate the role of mTORC2 in the MC903-induced skin inflammation. In order to delete the Rictor alleles, the Ert2Cre-*Rictor*^fl/fl^ (referred to as Rictor cKO) mice were treated with tamoxifen (Figure 4A). Then, 2 nmol of MC903 was applied to WT and Rictor cKO mice. In contrast to Raptor cKO mice, Rictor cKO mice were susceptible to MC903 treatment, and the skin inflammation developed comparably in both WT and cKO mice (Figure 4B). The shape of the ears and the expression levels of cytokines, such as IL-4 and TSLP (Figure 4C), were also indistinguishable between WT and cKO mice, implying that acute ablation of Rictor did not impair the functions of skin barrier and immune cells. 

To make sure the roles of Raptor in MC903 skin inflammation, we deployed two different mouse models: Ert2Cre-*Pten*^fl/fl^ (referred to as Pten cKO) and rapamycin. Consistent with the results of the Raptor cKO study, the ear inflammation induced by MC903 was enhanced in the Pten cKO mice (Figure 4D,E) and diminished after rapamycin treatment (Figure 4F,G). Then, we analyzed the cytokine expression in mice treated with rapamycin and found that IL-4 was downregulated, but TSLP was upregulated by rapamycin, as it was in Raptor cKO mice (Figure 4H), which led us to hypothesize that rapamycin could work differently in immune cells and keratinocytes and search for the downstream target in the regulation of TSLP expression.

### 2.4. Rapamycin Upregulates TSLP in Keratinocytes through a HIF Pathway

**Figure 5:**
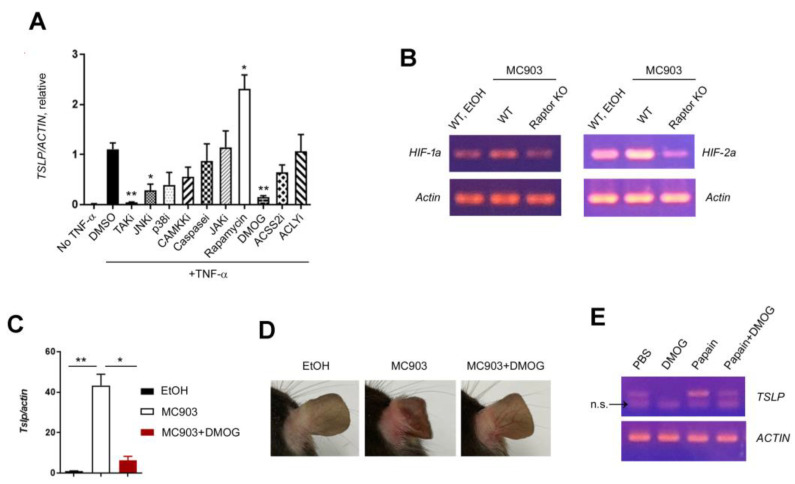
Rapamycin upregulated TSLP in keratinocytes. (**A**) qPCR shows mRNA expression of TSLP in HaCaT cells treated with TNF-α and diverse inhibitors. (**B**) RT-PCR shows the mRNA expression of HIF-1α and HIF-2α in ears treated with MC903. (**C**) qPCR shows the mRNA expression of TSLP in ears treated with MC903 and DMOG. (**D**) Gross appearance of the ears of WT mice treated with MC903 plus DMOG. (**E**) The gel electrophoresis picture of TSLP PCR in BEAS-2B cells treated with papain and DMOG. n.s. indicates ‘non-specific’. Data are presented as the mean ± SD. *, *p* < 0.05; **, *p* < 0.01.

To determine the downstream target of mTORC1 in the expression of TSLP, we treated HaCaT cells with TNF-α plus various inhibitors and performed the RT-qPCR analysis to quantify the amount of TSLP transcripts. Since the long form of TSLP mRNA contributes to the release of TSLP protein [27,28], we measured the long form of TSLP transcripts. Consistent with the previous reports [29] and in vivo data (Figure 4H), TSLP was upregulated by TNF-α, and the expression level of TSLP was higher in the presence of TNF-α and rapamycin. The expression of TSLP was decreased by the inhibitors of NF-κB (TAK), JNK, and PHD (DMOG) (Figure 5A). DMOG is a well-known inhibitor of PHD and helps to maintain HIF. Since HIF is a downstream target of mTORC1 as well as PHD, we hypothesized that the expression of TSLP was regulated by the mTORC1-HIF axis. To determine the relationship between mTORC1 and HIF, we examined the expression of HIF in WT and Raptor KO mice treated with MC903 and found that both HIF isoforms (HIF-1α and HIF-2α) were downregulated in Raptor KO skin (Figure 5B). Next, we applied DMOG on the ear together with MC903 and investigated whether DMOG reduced the expression of TSLP and MC903-induced skin inflammation in vivo. Not only the expression of TSLP (Figure 5C) but also the skin inflammation (Figure 5D) was reduced significantly by DMOG treatment. 

Papain is a protease known to cause occupational asthma [30] that also induces asthma-like inflammation in mice via TSLP [31], IL-33 [32], and ILC2 [33]. Since the mechanism of action of papain in lung inflammation seems to be like that of MC903 in the skin, we decided to check the effect of DMOG on the TSLP expression using BEAS-2B bronchial epithelial cell lines. Like the results in HaCaT cells, DMOG downregulated TSLP in BEAS-2B cells treated with papain (Figure 5E), supporting the idea that TSLP production under inflammatory conditions could be prevented by mTORC1-HIF signaling.

## 3. Discussion

In this study, we investigated the roles of mTORC1 and mTORC2 in an MC903-induced AD model and found that mTORC1 is essential for type 2 inflammation. Raptor (mTORC1) deficiency or rapamycin treatment dramatically reduced the expression of IL-4, inflammatory cell recruitment, and the extent of ear swelling. All of the results demonstrated the pro-inflammatory effect of mTORC1 on the development of AD. However, we also found an anti-inflammatory function of the mTORC1 signaling: TSLP was upregulated by Raptor deficiency or rapamycin treatment. Given that TSLP acts early in allergic inflammation, such as conditioning basophils [34] or dendritic cells [35], we speculated that the anti-inflammatory effect of rapamycin or Raptor deficiency gradually became predominant at the late stage of MC903-induced inflammation (via downregulation of IL-4) [36]. It has been reported that mTORC1 controls HIF signaling through various mechanisms at both transcription [37,38] and translation [39] levels. We also found that both HIF-1 and HIF-2 were downregulated in Raptor KO (Figure 5B), supporting the idea that mTORC1 regulates TSLP expression via HIF.

HIF and glycolysis have been reported to promote type 1 [40] and type 3 [41] inflammation. However, the role of HIF in type 2 inflammation remains unclear. Here, we found that DMOG inhibited the expression of TSLP (Figure 5B) and type 2 skin inflammation (Figure 5C), suggesting that HIF might reduce type 2 inflammation in the skin. Therefore, it would be intriguing to investigate whether HIF or DMOG inhibits type 2 inflammation without affecting type 1 or 3 inflammation. 

Lastly, we would like to mention the limitations of this study. In contrast to reactions in mice, MC903 did not upregulate TSLP in human keratinocytes [42], nor did it induce AD-like dermatitis in human skin. Instead, it inhibited human keratinocyte proliferation [43]. Therefore, extrapolation of our findings to human AD pathogenesis should be applied with great caution.

## 4. Materials and Methods

### 4.1. Mice

Wild-type (WT) C57BL/6 (B6) mice were purchased from Koatech. Raptor^fl/fl^, Rictor^fl/fl^, and Pten^fl/fl^ mice [44,45] were crossed with the tamoxifen-inducible Cre (Ert2Cre) transgenic mice (B6.129-Gt(ROSA)26Sor^tm1(cre/ERT2)Tyj/J^, The Jackson Laboratory). To delete floxed genes, tamoxifen (0.1 mg/ear, Sigma-Aldrich, St. Louis, MO, USA) was applied to the ear skin daily for 5 days. Genotyping was performed by using PCR or immunoblotting. All animal experimentations were conducted in accordance with guidelines and approval of the International Animal Care and Use Committees of Hallym University (Hallym 2020-28, Hallym 2021-59). 

### 4.2. MC903 Induced Murine AD Model

MC903 (calcipotriol, Sigma-Aldrich) was dissolved in EtOH and topically applied to mouse ears. Mice were sensitized with 2 nmol of MC903 for 7 days unless specified otherwise. As vehicle control, the same volume of EtOH was applied to mouse ears. During MC903 treatment, ear thickness was measured and recorded using a micrometer (Mitutoyo, Kawasaki City, Tokyo).

### 4.3. Tissue Preparation and Flow Cytometry

The ears were minced and digested in 2 mL HBSS containing 0.1 mg/mL DNase I and 0.1 mg/mL Liberase TL (Sigma-Aldrich) for 1 h at 37 °C. The suspension was then passed through a 70 μm cell strainer (SPL). For surface staining, the cells were stained with antibodies for 30 min at 4 °C in the dark. For intracellular staining, the cells were stained using Foxp3 Staining Buffer set (eBioscience, San Diego, CA, USA). The antibodies included anti-mouse CD11b BV510 (M1/70), CD11c BV421 (N418), CD3 FITC (145-2C11), CD4 PE/Cy7 (RM4-5), CD45 PE/Cy7 or BV510 (30-F11), CD8 APC (53-6.7), CD90.2 APC/Cy7 (30-H12), Ly-6G PerCP/Cy5.5 (1A8), Siglec-F A647 (E50-2440), TCRβ APC/Cy-7 (H57-597), TCRγ/δ PerCP/Cy5.5 (GL3), Gata-3 eFluor660 (TWAJ), and T1/ST2 biotinylated (DJ8) antibodies (all from Biolegend (San Diego, CA, USA), BD Biosciences (San Jose, CA, USA), eBioscience, and mdbioproducts (Oakdale, MN, USA)). Data were acquired through FACS Canto-II (BD Biosciences) and were analyzed with FlowJo software (version 10, BD Biosciences).

### 4.4. Cell Culture

The human keratinocyte HaCaT cells (ATCC) and human bronchial epithelial cell BEAS-2B (kindly provided by Professor Young-Hee Kang (Department of Food and Nutrition, Hallym University)) were used in this study. Both were cultured at 37 °C under a humidified atmosphere of 5% CO_2_ in 10% FBS/DMEM or 10% FBS-BEGM (Bronchial Epithelial Cell Growth Medium BulletKit™, Lonza, Basel, Switzerland), respectively. For all cell stimulation experiments, 2 × 10^5^ cells were seeded in each well of a 24-well plate. When cells were grown to 80% confluence, cells were stimulated with TNF-α and various inhibitors for 2 h and then harvested in Trizol (Invitrogen, Waltham, MA, USA) for RNA extraction. The inhibitors (all from Sigma-Aldrich, except ACSS2 and ACLY inhibitors) are as follows: TAK inhibitor, (5Z)-7-Oxozeaenol; JNK inhibitor, JNK Inhibitor II; p38 inhibitor, SB203580; CAMKK inhibitor, STO-609; Caspase inhibitor, Z-VAD-FMK; JAK inhibitor, Pyridone 6; ACSS2 inhibitor N-(2,3-di-2-thienyl-6-quinoxalinyl)-N′-(2-methoxyethyl) urea (Hit2Lead); ACLY inhibitor, and SB 204990 (Tocris, Bristol, UK).

### 4.5. Immunohistochemistry

Ear tissues were fixed in a 10% neutral-buffered formalin, dehydrated, and embedded in paraffin. Paraffin sections (5 μm thick) were blocked (10% normal goat serum in PBS) and incubated with anti-pS6 antibodies (Cell Signaling, Danvers, MA, USA) overnight at 4 °C. Bound primary antibodies were detected with HRP-Conjugate secondary antibodies and a DAB IHC detection kit (Abcam, Cambridge, UK). 

### 4.6. Quantitative PCR (qPCR)

RNA was extracted using Trizol (Thermo Fisher Scientific, Waltham, MA, USA) and reverse-transcribed into cDNA using QuantiTect Reverse Transcription kit (Qiagen, Hilden, Germany). All data were normalized to actin. Non-specific amplification was checked by using melting curves and agarose gel electrophoresis. The sequences of primers are as follows.

Mouse *Il4* Forward: 5′-ACAGGAGAAGGGACGCCA-3′

Mouse *Il4* Reverse: 5′-GAAGCCCTACAGACGAGCTCA-3′

Mouse *Tslp* Forward: 5′-AGGCTACCCTGAAACTGAG-3′

Mouse *Tslp* Reverse: 5′-GGAGATTGCATGAAGGAATACC-3′

Mouse *Il33* Forward: 5′-GGTGTGGATGGGAAGAAGCTG-3′

Mouse *Il33* Reverse: 5′-GAGGACTTTTTGTGAAGGACG-3′

Mouse *Il25* Forward: 5′-CAGCAAAGAGCAAGAACC-3′

Mouse *Il25* Reverse: 5′-CCTGTCCAACTCATAGC-3’

Mouse *Actin* Forward: 5′-CATCCGTAAAGACCTCTATGCCAAC-3′

Mouse *Actin* Reverse: 5′-ATGGAGCCACCGATCCACA-3′

### 4.7. Immunoblotting

The pieces of ear skin were lysed with cold radioimmunoprecipitation (RIPA) buffer containing a protease inhibitor cocktail (Roche, Basel, Switzerland). Subsequently, the collected protein was subjected to sodium dodecylsulfate (SDS)–polyacrylamide gel electrophoresis (PAGE) and blotted using nitrocellulose paper (Amershan). The membrane was probed with antibodies against Phospho-S6 Ribosomal Protein (Ser240/244) (D68F8, Cell Signaling Technology, Danvers, MA, USA) and Phospho-Akt (Ser473) (193H12, Cell Signaling Technology). GAPDH (D16H11, Cell Signaling Technology) was considered a loading control.

### 4.8. Statistical Analyses

A two-tailed, unpaired Student’s *t*-test was used to calculate the statistical significance of differences between groups. The *p* values are represented *** *p* < 0.001; ** *p* < 0.01; and * *p* < 0.05, whereas NS, not significant, is used to denote *p* values > 0.05. Error bars indicate s.d.

## Data Availability

The datasets used in the study are available from the corresponding author upon request.

## References

[1] Langan S.M., Irvine A.D., Weidinger S. (2020). Atopic dermatitis. Lancet.

[2] Napolitano M., Fabbrocini G., Martora F., Picone V., Morelli P., Patruno C. (2021). Role of Aryl Hydrocarbon Receptor Activation in Inflammatory Chronic Skin Diseases. Cells.

[3] Furue M., Hashimoto-Hachiya A., Tsuji G. (2019). Aryl Hydrocarbon Receptor in Atopic Dermatitis and Psoriasis. Int. J. Mol. Sci..

[4] Weidinger S., Beck L.A., Bieber T., Kabashima K., Irvine A.D. (2018). Atopic dermatitis. Nat. Rev. Dis. Prim..

[5] Dharmage S.C., Lowe A.J., Matheson M.C., Burgess J.A., Allen K.J., Abramson M.J. (2014). Atopic dermatitis and the atopic march revisited. Allergy.

[6] Binderup L., Bramm E. (1988). Effects of a novel vitamin D analogue MC903 on cell proliferation and differentiation in vitro and on calcium metabolism in vivo. Biochem. Pharmacol..

[7] Molin L., Cutler T.P., Helander I., Nyfors B., Downes N., Calcipotriol Study Group (1997). Comparative efficacy of calcipotriol (MC903) cream and betamethasone 17-valerate cream in the treatment of chronic plaque psoriasis. A randomized, double-blind, parallel group multicentre study. Br. J. Dermatol..

[8] Li M., Hener P., Zhang Z., Kato S., Metzger D., Chambon P. (2006). Topical vitamin D3 and low-calcemic analogs induce thymic stromal lymphopoietin in mouse keratinocytes and trigger an atopic dermatitis. Proc. Natl. Acad. Sci. USA.

[9] Li M., Hener P., Zhang Z., Ganti K.P., Metzger D., Chambon P. (2009). Induction of thymic stromal lymphopoietin expression in keratinocytes is necessary for generating an atopic dermatitis upon application of the active vitamin D3 analogue MC903 on mouse skin. J. Investig. Dermatol..

[10] Kim B.S., Siracusa M.C., Saenz S.A., Noti M., Monticelli L.A., Sonnenberg G.F., Hepworth M.R., Van Voorhees A.S., Comeau M.R., Artis D. (2013). TSLP elicits IL-33-independent innate lymphoid cell responses to promote skin inflammation. Sci. Transl. Med..

[11] Schwartz C., Eberle J.U., Hoyler T., Diefenbach A., Lechmann M., Voehringer D. (2016). Opposing functions of thymic stromal lymphopoietin-responsive basophils and dendritic cells in a mouse model of atopic dermatitis. J. Allergy Clin. Immunol..

[12] Kim B.S., Wang K., Siracusa M.C., Saenz S.A., Brestoff J.R., Monticelli L.A., Noti M., Wojno E.D.T., Fung T.C., Kubo M. (2014). Basophils promote innate lymphoid cell responses in inflamed skin. J. Immunol..

[13] Siracusa M.C., Saenz S.A., Hill D.A., Kim B.S., Headley M.B., Doering T.A., Wherry E.J., Jessup H.K., Siegel L.A., Kambayashi T. (2011). TSLP promotes interleukin-3-independent basophil haematopoiesis and type 2 inflammation. Nature.

[14] Leyva-Castillo J.M., Hener P., Michea P., Karasuyama H., Chan S., Soumelis V., Li M. (2013). Skin thymic stromal lymphopoietin initiates Th2 responses through an orchestrated immune cascade. Nat. Commun..

[15] Saxton R.A., Sabatini D.M. (2017). mTOR Signaling in Growth, Metabolism, and Disease. Cell.

[16] Liu G.Y., Sabatini D.M. (2020). mTOR at the nexus of nutrition, growth, ageing and disease. Nat. Rev. Mol. Cell Biol..

[17] Guertin D.A., Stevens D.M., Thoreen C.C., Burds A.A., Kalaany N.Y., Moffat J., Brown M., Fitzgerald K.J., Sabatini D.M. (2006). Ablation in mice of the mTORC components raptor, rictor, or mLST8 reveals that mTORC2 is required for signaling to Akt-FOXO and PKCalpha, but not S6K1. Dev. Cell.

[18] Guertin D.A., Guntur K.V., Bell G.W., Thoreen C.C., Sabatini D.M. (2006). Functional genomics identifies TOR-regulated genes that control growth and division. Curr. Biol..

[19] Semenza G.L. (2014). Oxygen sensing, hypoxia-inducible factors, and disease pathophysiology. Annu. Rev. Pathol..

[20] Delgoffe G.M., Pollizzi K.N., Waickman A.T., Heikamp E., Meyers D.J., Horton M.R., Xiao B., Worley P.F., Powell J.D. (2011). The kinase mTOR regulates the differentiation of helper T cells through the selective activation of signaling by mTORC1 and mTORC2. Nat. Immunol..

[21] Yang K., Shrestha S., Zeng H., Karmaus P.W., Neale G., Vogel P., Guertin D.A., Lamb R.F., Chi H. (2013). T cell exit from quiescence and differentiation into Th2 cells depend on Raptor-mTORC1-mediated metabolic reprogramming. Immunity.

[22] Ding X., Willenborg S., Bloch W., Wickstrom S.A., Wagle P., Brodesser S., Roers A., Jais A., Bruning J.C., Hall M.N. (2020). Epidermal mammalian target of rapamycin complex 2 controls lipid synthesis and filaggrin processing in epidermal barrier formation. J. Allergy Clin. Immunol..

[23] Karagianni F., Pavlidis A., Malakou L.S., Piperi C., Papadavid E. (2022). Predominant Role of mTOR Signaling in Skin Diseases with Therapeutic Potential. Int. J. Mol. Sci..

[24] Jung K.E., Lee Y.J., Ryu Y.H., Kim J.E., Kim H.S., Kim B.J., Kang H., Park Y.M. (2015). Effects of topically applied rapamycin and mycophenolic acid on TNCB-induced atopic dermatitis-like skin lesions in NC/Nga mice. Int. Immunopharmacol..

[25] Yang F., Tanaka M., Wataya-Kaneda M., Yang L., Nakamura A., Matsumoto S., Attia M., Murota H., Katayama I. (2014). Topical application of rapamycin ointment ameliorates *Dermatophagoides farina* body extract-induced atopic dermatitis in NC/Nga mice. Exp. Dermatol..

[26] Naeem A.S., Tommasi C., Cole C., Brown S.J., Zhu Y., Way B., Willis Owen S.A., Moffatt M., Cookson W.O., Harper J.I. (2017). A mechanistic target of rapamycin complex 1/2 (mTORC1)/V-Akt murine thymoma viral oncogene homolog 1 (AKT1)/cathepsin H axis controls filaggrin expression and processing in skin, a novel mechanism for skin barrier disruption in patients with atopic dermatitis. J. Allergy Clin. Immunol..

[27] Xie Y., Takai T., Chen X., Okumura K., Ogawa H. (2012). Long TSLP transcript expression and release of TSLP induced by TLR ligands and cytokines in human keratinocytes. J. Dermatol. Sci..

[28] Fornasa G., Tsilingiri K., Caprioli F., Botti F., Mapelli M., Meller S., Kislat A., Homey B., Di Sabatino A., Sonzogni A. (2015). Dichotomy of short and long thymic stromal lymphopoietin isoforms in inflammatory disorders of the bowel and skin. J. Allergy Clin. Immunol..

[29] Jeong H., Shin J.Y., Kim M.J., Na J., Ju B.G. (2019). Activation of Aryl Hydrocarbon Receptor Negatively Regulates Thymic Stromal Lymphopoietin Gene Expression via Protein Kinase Cdelta-p300-NF-kappaB Pathway in Keratinocytes under Inflammatory Conditions. J. Investig. Dermatol..

[30] Novey H.S., Marchioli L.E., Sokol W.N., Wells I.D. (1979). Papain-induced asthma—Physiological and immunological features. J. Allergy Clin. Immunol..

[31] Kabata H., Flamar A.L., Mahlakoiv T., Moriyama S., Rodewald H.R., Ziegler S.F., Artis D. (2020). Targeted deletion of the TSLP receptor reveals cellular mechanisms that promote type 2 airway inflammation. Mucosal Immunol..

[32] Oboki K., Ohno T., Kajiwara N., Arae K., Morita H., Ishii A., Nambu A., Abe T., Kiyonari H., Matsumoto K. (2010). IL-33 is a crucial amplifier of innate rather than acquired immunity. Proc. Natl. Acad. Sci. USA.

[33] Halim T.Y., Krauss R.H., Sun A.C., Takei F. (2012). Lung natural helper cells are a critical source of Th2 cell-type cytokines in protease allergen-induced airway inflammation. Immunity.

[34] Sokol C.L., Barton G.M., Farr A.G., Medzhitov R. (2008). A mechanism for the initiation of allergen-induced T helper type 2 responses. Nat. Immunol..

[35] Soumelis V., Reche P.A., Kanzler H., Yuan W., Edward G., Homey B., Gilliet M., Ho S., Antonenko S., Lauerma A. (2002). Human epithelial cells trigger dendritic cell-mediated allergic inflammation by producing TSLP. Nat. Immunol..

[36] Bapat S.P., Whitty C., Mowery C.T., Liang Y., Yoo A., Jiang Z., Peters M.C., Zhang L.J., Vogel I., Zhou C. (2022). Obesity alters pathology and treatment response in inflammatory disease. Nature.

[37] Land S.C., Tee A.R. (2007). Hypoxia-inducible factor 1alpha is regulated by the mammalian target of rapamycin (mTOR) via an mTOR signaling motif. J. Biol. Chem..

[38] He L., Gomes A.P., Wang X., Yoon S.O., Lee G., Nagiec M.J., Cho S., Chavez A., Islam T., Yu Y. (2018). mTORC1 Promotes Metabolic Reprogramming by the Suppression of GSK3-Dependent Foxk1 Phosphorylation. Mol. Cell.

[39] Dodd K.M., Yang J., Shen M.H., Sampson J.R., Tee A.R. (2015). mTORC1 drives HIF-1alpha and VEGF-A signalling via multiple mechanisms involving 4E-BP1, S6K1 and STAT3. Oncogene.

[40] Lee J.H., Elly C., Park Y., Liu Y.C. (2015). E3 Ubiquitin Ligase VHL Regulates Hypoxia-Inducible Factor-1alpha to Maintain Regulatory T Cell Stability and Suppressive Capacity. Immunity.

[41] Shi L.Z., Wang R., Huang G., Vogel P., Neale G., Green D.R., Chi H. (2011). HIF1alpha-dependent glycolytic pathway orchestrates a metabolic checkpoint for the differentiation of TH17 and Treg cells. J. Exp. Med..

[42] Landheer J., Giovannone B., Sadekova S., Tjabringa S., Hofstra C., Dechering K., Bruijnzeel-Koomen C., Chang C., Ying Y., de Waal Malefyt R. (2015). TSLP is differentially regulated by vitamin D3 and cytokines in human skin. Immun. Inflamm. Dis..

[43] Kragballe K., Wildfang I.L. (1990). Calcipotriol (MC 903), a novel vitamin D3 analogue stimulates terminal differentiation and inhibits proliferation of cultured human keratinocytes. Arch. Dermatol. Res..

[44] Lee K., Gudapati P., Dragovic S., Spencer C., Joyce S., Killeen N., Magnuson M.A., Boothby M. (2010). Mammalian target of rapamycin protein complex 2 regulates differentiation of Th1 and Th2 cell subsets via distinct signaling pathways. Immunity.

[45] Lee K., Nam K.T., Cho S.H., Gudapati P., Hwang Y., Park D.S., Potter R., Chen J., Volanakis E., Boothby M. (2012). Vital roles of mTOR complex 2 in Notch-driven thymocyte differentiation and leukemia. J. Exp. Med..

